# Evaluation of efficacy and safety of *Qiangzhu-qinggan* formula as an adjunctive therapy in adult patients with severe influenza: study protocol for a randomized parallel placebo-controlled double-blind multicenter trial

**DOI:** 10.1186/s13063-021-05929-8

**Published:** 2021-12-27

**Authors:** Lei Qiu, Xian-wei Wu, Shao-yan Zhang, Ming Yang, Shun-xian Zhang, Ji-you Fu, Cui Li, Zhi-jie Zhang, Pei-yong Zheng, Zhen-hui Lu

**Affiliations:** 1 Institute of Respiratory Diseases, Longhua Hospital Affiliated to Shanghai University of Traditional Chinese Medicine, No.725 South Wanping Road, No.7 building, Xuhui District, Shanghai, People’s Republic of China; 2 Department of Epidemiology, School of Public Health, Fudan University, 130 Dongan Road, No.8 building, Xuhui District, Shanghai, People’s Republic of China

**Keywords:** Severe influenza, Adjunctive therapy, *Qiangzhu-qinggan* formula, Randomized controlled trial, Traditional Chinese medicine

## Abstract

**Background:**

Influenza can fall into three categories according to severity: mild influenza, severe influenza, and critical influenza. Severe influenza can result in critical illness and sometimes death particularly in patients with comorbidities, advanced age, or pregnancy. Neuraminidase inhibitors (NAIs) are the only antiviral drugs in widespread use for influenza. However, the effectiveness of NAIs against severe influenza is uncertain. New effective drugs or regimens are therefore urgently needed. *Qiangzhu-qinggan (QZQG)* formula has been found to be effective against influenza virus infection during long-term application in China, which lacks support of evidence-based clinical trial till now. This study is designed to assess the efficacy and safety of *QZQG* formula as an adjuvant therapy in adult patients with severe influenza.

**Methods:**

This protocol is drawn up in accordance with the SPIRIT guidelines and CONSORT Extension for Chinese herbal medicine formulas. This is a randomized, placebo-controlled, double-blind, multicenter trial. Two hundred twenty-eight adults with severe influenza are randomly assigned in a 1:1 ratio to *QZQG* or placebo for 7 days. All participants need to receive 1 day of screening before randomization, 7 days of intervention, and 21 days of observation after randomization. The primary outcome is the proportion of clinical improvement, defined as the proportion of patients who met the criteria of 3 points or less in the seven-category ordinal scale or 2 points or less in National Early Warning Score 2 within 7 days after randomization.

**Discussion:**

This is the first randomized, controlled, parallel, double-blind clinical trial to evaluate the efficacy and safety of traditional Chinese herbal formula granules as an adjuvant therapy in adult patients with severe influenza. This study aims to redefine the value of traditional Chinese herbal medicines in the treatment of virus-related respiratory infectious diseases and serves as an example of evidence-based clinical trials of other Chinese herbal medicines.

**Supplementary Information:**

The online version contains supplementary material available at 10.1186/s13063-021-05929-8.

## Introduction

### Background and rationale {6a}

“Spanish flu” pandemic in 1918 was described as the most devastating viral pandemic in history caused by an H1N1 influenza A virus that infected over 500 million and killed between 50 and 100 million people [1]. Influenza infection remains serious today. The Centers for Disease Control and Prevention (CDC) estimated that influenza virus infection caused more than 35.5 million illnesses, more than 16.5 million medical visits, 490,600 hospitalizations, and 34,200 deaths during the 2018–2019 influenza season in the USA [2]. It is estimated that an annual mean of 88,100 (95% CI 84,200–92,000) influenza-associated excess respiratory deaths occurred in China during the 2010–2011 through 2014–2015 seasons, corresponding to 8.2% (95% CI 7.9–8.6) of all reported respiratory deaths [3]. Seasonal influenza worldwide is estimated to cause 291,243–645,832 influenza-associated respiratory deaths annually [4]. Although mostly a self-limited disease, 5–10% of hospitalized patients with influenza required ICU admission mainly because of acute respiratory failure [5]. Influenza can be divided into three categories according to severity: mild influenza, severe influenza, and critical influenza [6]. Severe influenza is one of the main causes of critical illness and death particularly in patients with co-morbidities, advanced age, or pregnancy [4].

Antiviral drugs currently approved for use in China, the USA, and the European Union include the neuraminidase inhibitors (NAI) and adamantane derivatives [6–8]. Currently circulating seasonal influenza viruses are resistant to the adamantane derivatives and so the use of these agents is not recommended [7, 8]. It is advised that clinicians should start antiviral treatment in severe influenza as soon as possible with a single neuraminidase inhibitor (either oral oseltamivir, inhaled zanamivir, or intravenous peramivir) [9]. Baloxavir marboxil (XofluzaTM; baloxavir), a novel, cap-dependent endonuclease inhibitor, received its first global approval in Japan on 2018 for the treatment of influenza A or B virus infections in pediatric and adult patients [10]. However, adequate evidence-based clinical trials have not been conducted to define which antiviral drug severe influenza can benefit from [11]. As recommended by the WHO’s Global Influenza Strategy for 2019–2030, it is urgent to develop better treatment regimen for influenza [12].

A large amount of clinical evidence confirmed the efficacy and safety of Traditional Chinese medicine (TCM) in viral respiratory infections. As a major adjuvant therapy, TCM has been widely used to prevent and treat virus-related respiratory infection and included in the recommended treatment regimens for influenza in China [6]. A prospective, nonblinded, randomized controlled trial (RCT) in 2011 found that time to fever resolution was reduced by 19% (CI, 0.3% to 34%; *P* = 0.05) with oseltamivir plus *maxingshigan–yinqiaosan* compared with oseltamivir [13]. A RCT including 244 patients with influenza A virus infection in China has shown that *Lianhuaqingwen* capsule significantly reduced the severity of illness and the duration of symptoms including fever, cough, sore throat, and fatigue (*P* < 0.05) [14]. In a meta-analysis of 31 RCTs including 5514 cases of influenza [15], the authors concluded that TCM had significantly increased clinical efficacy compared with placebo or no intervention (93.46% vs. 79.03%, respectively; odds ratio, 3.99 [95% CI, 3.32 to 4.78]; *P* < 0.001), and no serious adverse effects were reported.

The mechanisms of TCM in treating influenza may include both antiviral and immunomodulatory effect. Numerous herbal extracts were shown to have antiviral effects against the influenza virus with multiple herbal antiviral targets mainly including hemagglutinin, neuraminidase, and matrix 2 proteins, which possess the ability to inhibit resistant influenza strains and have great application potential and scientific value for addressing the problem of emerging influenza antiviral resistance [16]. Many herbs exhibit beneficial immunomodulatory effects for the rapid recovery of viral infections [17, 18]. It is reported that *Jiawei-Yupingfeng-Tang* can alleviate influenza-induced lung lesions with both antiviral and immunomodulatory activity [19]. *Lianhuaqingwen* capsule was shown to exert broad-spectrum effects on a series of influenza viruses, including the newly emerged H7N9, and particularly regulates the immune response of virus infection [20].

*QZQG* formula is the combination and innovation of two ancient TCM formulas including *Jiuweiqianghuo* Decoction and *Zhuyeshigao* Decoction, which have been used in treating exogenous febrile disease (acute upper or lower respiratory tract infection) for hundreds of years. Our preliminary data show that the *QZQG* formula may improve influenza-like symptoms, shorten the course of disease, and reduce the proportion of critical illness (data not shown). However, no data are available from large-scale randomized controlled trials on the efficacy of *QZQG* and its adverse effects. Therefore, we aim to perform the first double-blinded, randomized, placebo-controlled trial to make clear the efficacy and safety of *QZQG* in adults with severe influenza. In the global context of COVID-19 pandemic, this trial will help to understand the value of traditional Chinese medicine as adjuvant therapy in severe and critically ill patients with respiratory viral infectious diseases such as influenza and COVID-19.

## Objectives {7}

Empirical evidence based on long-term clinical application shows that *QZQG* formula can effectively resist influenza virus infection. However, there are currently no reliable data from evidence-based clinical trials. We aim to carry out this trial to evaluate the efficacy and safety of QZQG formula plus western medicine regimen compared with placebo plus western medicine regimen in adults with severe influenza.

## Trial design {8}

This study was designed as a multicenter, randomized, parallel, double-blind, placebo-controlled trial. Eligible participants will be randomly divided into two groups at a 1:1 ratio: *QZQG* group and placebo group. Randomly assigned numbers will be provided by a third-party research institution (School of Public Health, Fudan University, China). All enrolled patients will receive 1 day of screening, 7 days of intervention, and 21 days of observation.

## Methods: participants, interventions, and outcomes

### Study setting {9}

**Fig. 1:**
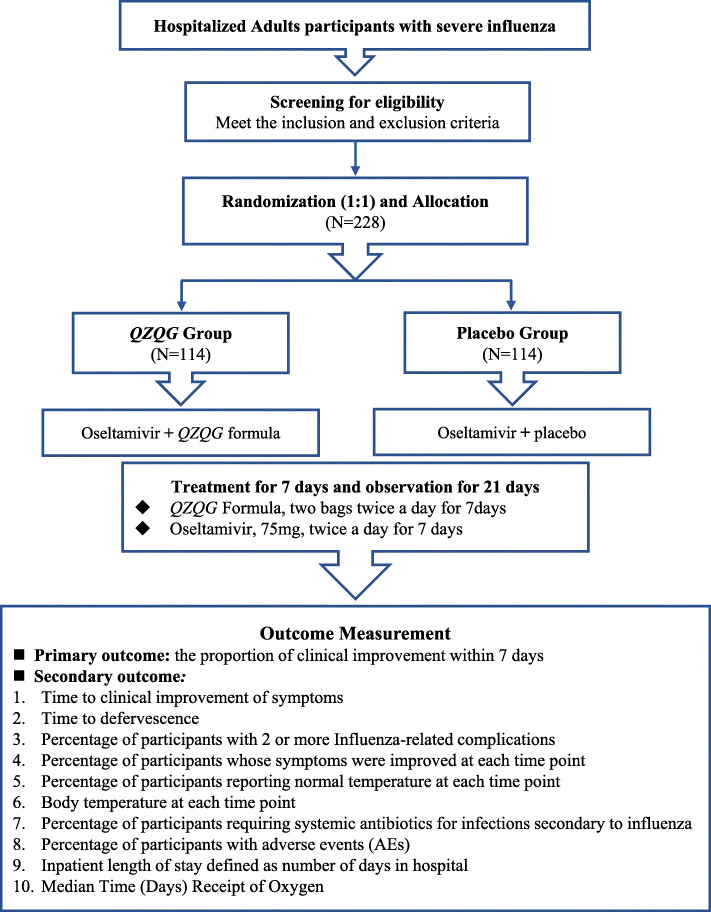
Study flowchart. Participants with severe influenza will be recruited, and they will be assigned randomly into two different groups. All participants will accept the 1-day screening, 7-day intervention, and 21-day observation. The data were collected to determine the efficacy and safety of *QZQG* Formula as an adjuvant therapy

This trial will be conducted in Longhua Hospital Affiliated to Shanghai University of Traditional Chinese Medicine, Huashan Hospital Affiliated to Fudan University, The First Affiliated Hospital of Anhui University of Traditional Chinese Medicine, Hefei Infectious Disease Hospital, Jiangsu Provincial Hospital of Traditional Chinese Medicine, Jiangsu Provincial Hospital for Infectious Diseases, and Zhejiang Provincial Hospital of Traditional Chinese Medicine. Prior to the trial, all personnel are trained in Longhua Hospital to ensure that the physicians and staff participating in the trial at every center fully understand all aspects of the trial (Fig. 1).

### Eligibility criteria {10}

Male and female patients with severe influenza aged 18–65 years are eligible for study participations. The diagnostic criteria for severe influenza referred to the “Scheme for diagnosis and treatment of influenza” issued by the National Health Commission of China in 2020 [6]. Patients will be enrolled if they conform to all inclusion criteria. However, they will not be enrolled if they have one of the exclusion criteria, rejection criteria, or termination standards.

#### Inclusion criteria

*The inclusion criteria are as follows:*

1. Diagnosis of influenza A and/or B by a positive Rapid Influenza Diagnostic Test (RIDT) or reverse transcriptase-polymerase chain reaction (RT-PCR).
  • Positive results from local tests are acceptable if conducted within the 24 h prior to screening.
  • A patient with a negative RIDT may be enrolled if influenza is suspected based on local surveillance data or if the patient reports contact with a known case of influenza within the prior 7 days and all other inclusion criteria are met.
2. Granting of written informed consent.
3. Male or female aged 18 to 65 years at the time of signing informed consent form.
4. Ability to comply with the study protocol, in the investigator’s judgment.
5. Participants who require hospitalization for severe influenza or acquire influenza during hospitalization, the severity of which requires an extension of hospitalization.
6. Participants will require at least one of the following objective criteria of severe influenza: (i) duration with axillary temperature over 39 °C ≥ 3 days, accompanied by severe cough, sputum, blood sputum, or chest pain; (ii) rapid respiratory rate (≥30 times per minute), difficulty breathing and cyanosis of lips; (iii) arterial oxygen saturation (SaO2)/ pulse oxygen saturation (SPO2) ≤94% in room air condition; (iv) requires ventilation or supplemental oxygen to support respiration; (v) with only one complication related to influenza (e.g., pneumonia, central nervous system involvement, myositis, rhabdomyolysis, acute exacerbation of chronic kidney disease, asthma or chronic obstructive pulmonary disease (COPD), severe dehydration, myocarditis, pericarditis, exacerbation of ischemic heart disease).
7. The time interval between the onset of symptoms and randomization is within 96 h. The onset of symptoms is defined as either time of the first increase in body temperature (≥ 38 °C) or time when the patient experiences at least one general or respiratory symptom.
8. Conforming to the standard of TCM syndrome differentiation.

#### Exclusion criteria

*The exclusion criteria are as follows:*

1. Participants hospitalized for exclusively social reasons (e.g., lack of caregivers at home).
2. Participants expected to be discharged within 48 h, according to the investigator’s judgment.
3. Participants weighing < 40 kg.
4. Participants who have received more than 48 h of antiviral treatment for the current influenza infection prior to screening.
5. Participants with known severe renal impairment or receiving continuous renal replacement therapy, hemodialysis, peritoneal dialysis.
6. Participants with any of the following laboratory abnormalities detected within 24 h prior to or during screening (according to local laboratory reference ranges:
  • Alanine transaminase (ALT) or aspartate transaminase (AST) level > 5 times the upper limit of normal (ULN).
  • ALT or AST > 3 times the ULN and total bilirubin level > 2 times the ULN.
7. Any serious medical condition or abnormality in clinical laboratory tests that, in the investigator’s judgment, precludes the participant’s safe participation in and completion of the study.
8. With mental changes or convulsions (e.g., slow response, drowsiness and restlessness).
9. With severe gastrointestinal symptoms (e.g., severe nausea, vomiting, diarrhea and even dehydration).
10. With one of the following critical illnesses: respiratory failure, acute necrotic encephalopathy, septic shock, multiple organ dysfunction, or other serious clinical conditions requiring intensive care.
11. With tuberculosis, measles, AIDS, or other infectious diseases.
12. Women who are pregnant (including a positive pregnancy test at enrollment), breastfeeding, or within 2 weeks post-partum.
13. Known history of allergy or severe intolerance of oseltamivir and herbal medicines, as determined by the investigator.
14. Currently or have been involved in another anti-influenza treatment trial in the last 28 days.
15. Patients who have been treated with oral Chinese medicine within 4 weeks.
16. Patients who, in the opinion of the investigator, would be unlikely to comply with required study visits, self-assessments, and interventions.

### TCM syndrome differentiation criteria

According to the TCM theory, *QZQG* formula is suitable for severe influenza that meets the pattern of Plague Simultaneously Involving Weifen and Qifen (*PSIWF* pattern). The TCM differentiation criteria for *PSIWF* pattern include symptoms, signs for the tongue, and signs for the pulse. The symptoms include at least high fever, cough, sticky phlegm, thirsty, sore throat, and red eyes. The signs for the tongue are red tongue and thick yellow or greasy tongue. The signs for the pulse are slippery or rapid pulse. To meet the criteria of TCM syndrome differentiation, participants should have all symptoms of *PSIWF* pattern as well as the TCM signs for the tongue and pulse.

### Informed consent {26a}

A doctor will confirm if the patients satisfy the criteria after consent being given. Subsequently, the medical officers of the trial team in every center will communicate with the patients and their families to inform the possible benefits and risks of participating in this study. Patients will be given sufficient time to fully consider trial entry and to ask questions of investigators. Patients willing to participate in this trial need to sign a written informed consent form. A consent for clinical biological specimens will also be obtained to specifically address the collection of biological specimens for future use including serum, plasma, urine, and fecal specimens.

### Interventions

#### Explanation for the choice of comparators {6b}

The active arm is orally administered *QZQG* formula in addition to routine supportive care, in which oseltamivir is the only antiviral allowed. *QZQG* formula has been used in treating exogenous febrile disease (acute upper or lower respiratory tract infection) in China for hundreds of years. *Jiuweiqianghuo* granule has been recommended by the guideline for the diagnosis and treatment of influenza in China [6]. There are safety and efficacy data for *QZQG* formula in individuals with respiratory tract infection [21, 22]. The dose of *QZQG* was selected on long-term clinical application experience in China. The control arm is placebo in addition to routine supportive care.

#### Intervention description {11a}

*The intervention is described as follows for the experimental and control groups:*

The *QZQG* group will receive two bags of *QZQG* formula granules (4.6 g/per bag), twice a day, 30 min after eating for 7 days. Whole ingredients of *QZQG* formula include *Rhizoma seu Radix Notopterygii* (*Qiang Huo*) 18g, *Gypsum Fibrosum* (*Sheng Shi Gao*) 25g, *Codonopsis Radix* (*Dang Shen*) 7.5g, *Angelicae Dahuricae Radix* (*Bai Zhi*) 18g, *Saposhnikovia Divaricata* (*Fang Feng*) 18g, *Rhizoma Atractylodis* (*Fu Chao Cang Zhu*) 18g, *Pinellia Ternata* (*Ban Xia*) 9g, *Taraxacum* (*Pu Gong Ying*) 15g, *Astragalus Membranaceus* (*Huang Qi*) 15g, *Lophatherum Gracile *(*Dan Zhu Ye*) 3g, *Radix Ophiopogonis* (*Mai Dong*) 15g, *Rehmannia Glutinosa* (*Sheng Di Huang*) 12g, *Scutellaria baicalensis* (*Huang Qin*) 12g, *Asari Radix Rhizoma* (*Xi Xin*) 3 g, *Ligusticum wallichii* (*Chuan Xiong*) 12g, *Honey-fried Herba Ephedrae*(*Zhi Ma Huang*) 6g, *Vitex negundo*(*Huang Jing Zi*) 20g and *Clycyrrhizae Radix Et Rhizoma* (*Gan Cao*) 12g. *QZQG* formula granules and placebo granules are manufactured and provided by Sichuan New Green Pharmaceutical Technology Development Co., Ltd. The Chinese herbal medicine placebo with the same weight as the test drugs consists of 20-fold dilution of 4.60 g of *QZQG* formula granules and maltodextrin with the addition of artificial pigment and flavoring agents.

The placebo group will receive placebo granules and used the same medication as the test group. Placebo is identical to the test drug in appearance, smell, and taste. This preparation of placebo has been frequently used in herbal medicine trials in China. All drugs are hidden in a uniform package with the same label, and each package contains a 7-day dose. Both test drugs and placebo will be packaged and labeled by School of Public Health, Fudan University, according to the random number table and drug blinds.

#### Criteria for discontinuing or modifying allocated interventions {11b}

Use of study drug will be halted or modified if any of the following criteria develop:

1. Severe gastrointestinal symptoms prevented oral administration of the trial drug.
2. Respiratory compromise: dyspnea, wheezing, stridor, hypoxemia.
3. A decrease in systolic blood pressure to < 90 mmHg or > 30% decrease from baseline or a diastolic drop of > 30% from baseline.
4. Tachycardia with an increase in resting heart rate to > 130 bpm; or bradycardia < 40 that is associated with dizziness, nausea, or feeling faint.
5. Syncope.
6. Confusion.
7. Any other symptom or sign which, in the investigators’ judgment, requires withdrawal of the subject from the study warrants halting the oral administration.

#### Strategies to improve adherence to interventions {11c}

All participants are inpatients and receive the whole process management of the research doctor, which will significantly improve the compliance. In every site, an independent drug administrator is responsible for dispensing, reclaiming, storing, and recording of all test drugs. The research nurses in each center will be responsible for the distribution of drugs and placebo and fill in the drug management diary. The subjects will complete the medication under the direct supervision of nurses.

#### Relevant concomitant care permitted or prohibited during the trial {11d}

Considering ethical requirements of IRB, all enrolled patients need to receive one oseltamivir capsule (75 mg/per capsule), twice a day for 7 days [6]. Since patients with severe influenza in the all centers of China are hospitalized, the necessary intravenous fluid therapy is allowed. When the body temperature of a participant rises to higher than 39.0 °C, physical cooling and antipyretics are allowed to be used to reduce the fever. The treatments for hypertension, diabetes, cerebral infarction, and other underlying diseases are allowed as well. The doctors of the trial team must carefully record the name, dose, usage, and duration of the concomitant drugs or treatment in the case report form (CRF).

However, no other antivirals for influenza are allowed during the study period. Adjunctive therapies such as corticosteroids were prohibited. In addition, it is absolutely forbidden to take other Chinese herbal medicines and Chinese patent medicine during the study period or within 14 days before randomization. When the patients have drug-related adverse events or serious complications such as viral pneumonia, hepatoprotective drug, antibacterials, and other necessary treatments are completely allowed and recorded.

### Outcomes measurements {12}

#### Primary outcome measure

The primary outcome is proportion of clinical improvement within 7 days. And clinical improvement is defined as at least one of the following criteria: (i) 3 points or less in the 7-category ordinal scale; (ii) National Early Warning Score 2 (NEWS2) of ≤2 and maintained for 24 h [23]. Seven-category ordinal scale was used in other influenza study [24–27]. The seven-category ordinal scale consisted of the following categories: 1, not hospitalized with resumption of normal activities; 2, not hospitalized, but unable to resume normal activities; 3, hospitalized, not requiring supplemental oxygen; 4, hospitalized, requiring supplemental oxygen; 5, hospitalized, requiring nasal high-flow oxygen therapy, noninvasive mechanical ventilation, or both; 6, hospitalized, requiring ECMO, invasive mechanical ventilation, or both; and 7, death. The UK’s National Early Warning Score 2 have good predictive abilities in patients with infections and sepsis [23], which are detailed in the Table 1.

**Table 1:**
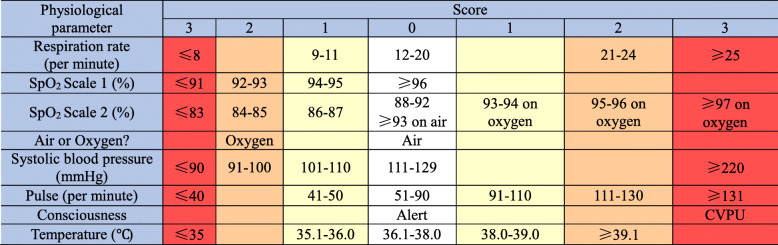
National Early Warning Score 2 (NEWS2)

NEWS2: (i) The oxygen saturation should be scored according to either the SpO2 Scale 1 or 2 presented in the table above. The SpO2 Scale 2 is for patients with a target oxygen saturation requirement of 88–92% (e.g., in patients with hypercapnic respiratory failure related to advanced lung diseases, such as chronic obstructive pulmonary disease [COPD]). This should only be used in patients confirmed to have hypercapnic respiratory failure by blood gas analysis on either a prior or their current hospital admission. The decision to use the SpO2 Scale 2 should be made by the treating physician and should be recorded in the eCRF. In all other circumstances, the SpO2 Scale 1 should be used. (ii) For physiological parameter “Air or Oxygen?”: Any patients requiring the use of oxygen or other forms of ventilation to maintain oxygen saturations and support respiration should be assigned a score of 2. (iii) The consciousness level should be recorded according to the best clinical condition of the patient during the assessment. Patients who are assessed as “Alert” (A) should be assigned a score of 0. Patients assessed as “New Confusion” (C), “Responsive to Voice” (V), “Responsive to Pain” (P), or “Unconscious” should be assigned a score of 3

#### Secondary outcome measure

Secondary outcomes are the following:

1. Time to clinical improvement of symptoms (TTCIS) [time frame: baseline to day 14].
  Participants assessed the severity of 7 influenza-associated symptoms (cough, sore throat, headache, nasal congestion, feverishness/chills, muscle/joint pain, and fatigue) on a 4-point scale (0 = no symptoms, 1 = mild, 2 = moderate, and 3 = severe). TTCIS was defined as the time from the start of treatment to the time when all influenza symptoms were alleviated, as defined below, for a duration of at least 21.5 h:
  • Pre-existing symptoms (cough, fatigue, or muscle/joint pain that existed prior to influenza) that were worse at baseline must have improved at least 1 point from baseline.
  • New symptoms must have alleviated, defined as a symptom score of none (0) or mild (1).
2. Time to defervescence (TTD) [time frame: baseline to day 14].
  Time to defervescence includes defervescence onset time and the time to complete defervescence. Defervescence onset time is defined as the time from the start of treatment to the time when the body temperature decreases to ≤37.3 °C. And complete defervescence is defined as the time from the start of treatment to the time when the temperature of ≤37.3 °C sustained for ≥ 24 h [14, 28].
3. Percentage of participants with 2 or more Influenza-related complications [time frame: baseline to day 28].
  Secondary influenza complications include pneumonia, encephalitis, meningitis, encephalopathy, myelitis, Guillain-Barré syndrome, myocarditis, pericarditis, myositis, and septic shock.
4. Percentage of participants whose symptoms were improved at each time point [time frame: 12, 24, 36, 48, 72, 96, 120, 144, and 168 h after the initial dose of study treatment]. The criteria for improvement of influenza symptoms have been described above.
5. Percentage of participants reporting normal temperature at each time point [time frame: 12, 24, 36, 48, 72, 96, 120, 144, and 168 h after the initial dose of study treatment]. Defined as the percentage of participants whose axillary body temperature dropped to equal or less than 37.3 °C after the initiation of the study treatment.
6. Body temperature at each time point [time frame: 12, 24, 36, 48, 72, 96, 120, 144, and 168 h after the initial dose of study treatment]. Participant’s self-measured axillary temperature using an electronic thermometer.
7. Percentage of participants requiring systemic antibiotics for infections secondary to influenza [time frame: day 2 to day 28].
8. Percentage of participants with adverse events (AEs).
9. Inpatient length of stay defined as number of days in hospital [time frame: day 2 to day 28].
10. Median time (days) receipt of oxygen [time frame: day 2 to day 28].

### Participant timeline {13}

The participant timeline is shown in Table 2.

**Table 2:** shows the timeline of participant assessments/interventions

Process	Screening stage	Intervention stage	Observation stage		
	Day 0	Day 1 to day 7	Day 14 ± 1	Day 21 ± 2	Day 28 ± 2
**Acquisition of basic medical history**					
▪ Symptoms and signs	**x**				
▪ Basic conditions	**x**				
▪ Informed consent	**x**				
▪ Inclusion and exclusion criteria	**x**				
**Efficacy and safety assessment**					
▪ Vital signs (heart rate, blood pressure, breathing rate and oxygen saturation)	**x**	**x**	**x**	**x**	**x**
▪ Electrocardiograph	**x**	a			**x**
▪ Chest radiograph	**x**	a			**x**
▪ Laboratory inspection	**x**	a			**x**
▪ Record adverse events	**x**	**x**	**x**	**x**	**x**
▪ Body temperature	**x**	**x** (Measured at 4, 8, 12, 16, 20 and 24 o'clock)	**x**	**x**	**x**
▪ Record influenza symptoms	**x**	**x**	**x**	**x**	**x**
▪ Use of antibiotics		**x**	**x**	**x**	**x**
**Interventions**					
▪ *QZQG* formula or placebo		**x**			
▪ Concomitant medications (record only)	**x**	**x**	**x**	**x**	**x**
**Other**					
▪ Distribute patient information diary card	✓				
▪ Compliance record		✓			
▪ Inspection results record	✓	✓	✓	✓	✓
▪ Complete original record in CRF					✓
▪ CRF reviews and data entry					✓

×, must implement; a, necessary to implement

### Sample size {14}

Sample size calculations are based on the primary endpoint (the proportion of clinical improvement within 7 days after randomization). At the 5% significance level, a total of 101 patients per group is required to achieve 90% power and to determine an increase of 20% in the proportion of clinical improvement between *QZQG* group and the placebo group, assuming that the clinical improvement rate within 7 days after randomization of the placebo group receiving oseltamivir plus routine symptomatic treatment is about 50%. With an estimated dropout rate of 10–20%, a total of 228 patients are planned to be enrolled. If the initial hypothesis is not rejected, subsequent comparison is considered exploratory, and no conclusion is made.

### Recruitment {15}

Patients will be recruited from 10 clinical centers most of which are the designated hospitals for patients with severe influenza.

## Assignment of interventions: allocation

### Sequence generation{16a}, concealment {16b}, and implementation {16c}

According to a stratified block randomization method, all patients are stratified by study center and randomly assigned to the *QZQG* group and placebo group in a 1:1 ratio. The random sequence and the randomization list of every center will be generated by the independent specialist from School of Public Health, Fudan University, China using the PRCO PLAN function of the analysis system of SAS software (SAS, Cary, NC, USA). The randomization sequence will be concealed in a lightproof sealed envelope for emergency unblinding events, which will be kept by the independent specialist and the Sponsor. An independent drug administrator at every center will assign numbered packs of study drugs to eligible patients in order by randomization list.

An emergency letter including the random sequence and assignment has been prepared in all of centers. In any emergency medical situation, such as serious adverse event or deteriorative condition, the unblinding process will be started after contacting the Sponsor and the primary investigator. The investigators from Longhua Hospital Affiliated to Shanghai University of traditional Chinese Medicine will record the details of urgent unblinding and make sure the corresponding patient is excluded. If the rate of drug blinding leakage or emergency letter opening rises more than 20%, this trial will be considered as failure.

## Assignment of interventions: blinding

### Who will be blinded after assignment to interventions? {17a}

This is a double-blind trial in which participants, investigators, care providers, outcome assessors, and data analysts are all blinded. Assignment of interventions will only be unblinded after database lock.

### Circumstances and procedure for unblinding if needed {17b}

Unblinding is permissible in emergency situations when investigators believe that it is necessary to perform any agent treatment/action for the trial patient’s safety. To allow emergency unblinding the investigator will be supplied with a set of emergency envelopes. If emergency unblinding of a patient is necessary, an independent pharmacist opens the sealed envelope and informs the investigators of the allocation. And the pharmacist has to enter date and time as well as his/her name and signature on the unblinding form contained in the envelope. Information about the date, time, and reason of unblinding will be recorded in Clinical Case Forms (CRFs) and the envelope.

## Data collection methods

### Plans for assessment and collection of outcomes {18a}

Training sessions on outcome evaluation and data collection will be held for investigators from all centers prior to the start of the study. Investigators are responsible for assessment and collection of outcome, baseline, and other trial data, which are double checked by the data managers and clinical research associates. Nasopharyngeal/oropharyngeal swabs, lower respiratory tract specimens (sputum/tracheal aspirate/bronchial alveolar lavage fluid), and feces/anal swabs will be sent to a central laboratory, where tests will be performed according to laboratory standard operating procedures (SOPs). Data collection forms can be found at http://longhua.site. Three scales (seven-category ordinal scale, the severity scale of 7 influenza-associated symptoms and NEWS2) are used for the outcome measures.

### Plans to promote participant retention and complete follow-up {18b}

During the course of the study, participants may voluntarily withdraw from the trial for subjective or objective reasons at any time. When the subjects request to withdraw, the investigators at study sites must fully inform the importance of continuing to retain and complete the trial, including better observation of drug efficacy and potential adverse reactions, and providing more valuable data for follow-up studies.

## Data management {19}

The doctors in the trial teams will receive training through on-site meetings and remote video to fully understand the protocol and standard operating procedures of this clinical trial. Personnel who participate directly in this trial follow guidelines for Good Clinical Practice (GCP) to ensure the safety of patients, blinding of the study design, data quality, and adherence to the protocol. During the study, an independent Steering Committee will review and supervise all the original documents and CRFs. The PI and medical officers from Longhua Hospital will regularly audit the protocol compliance and enrollment progress through site visits or remote video conference to ensure compliance with the protocol and the data quality at every center. The essential documents (signed informed consent form, follow-up management, CRFs, record of adverse events, number and proportion of missed visits and losses to follow-up) will be monitored and checked for accuracy and completeness by the monitors monthly.

All CRFs will be reviewed by the investigators and independent Steering Committee. The completed CRFs will be securely stored in a locked location and finally sent to two independent data administrators from School of Public Health, Fudan University, who will be responsible for data inspection, entry, and management. When the trial is completed, the Principal Investigator, Sponsor, data administrators, and statisticians will perform a blind review to confirm the dataset. The final database will be locked and analyzed in line with the statistical analysis plan.

## Statistical methods

### Statistical methods for analyzing primary and secondary outcomes {20a}

The primary study analysis will occur when the last patient has either withdrawn or completed day 28 visit, and will be based on cleaned data for all patients up to and including this point. The clinical data will be managed and analyzed by the independent statisticians from a third party (School of Public Health, Fudan University,) in accordance with the statistical analysis plan. Data management and analysis will use SAS V.9.4 (SAS Institute, Cary, NC, USA).

All statistical inferences will be determined using two-sided tests. *P* < 0.05 will be considered significant, and 95% confidence intervals will be used. Data will be described as the mean, standard deviation, and confidence intervals. If necessary, minimum, maximum, P25, P75, and median values will be provided. Paired measurement data will also show the differences between the mean and standard deviation. When using nonparametric methods, median and mean values will be provided. Count data will be described using the frequency distribution and corresponding proportion. Qualitative information will be described using the positive rate, the number of positive cases, and the denominator.

Baseline data analyses (in FAS and PPS) will include demographic indicators and primary and secondary outcomes before intervention. Measurement data will be described using a *t*-test or *t*’ test (if the variance is absent). Count data will be described using the Pearson’s *χ*^2^ test. Rating data will be described using the two-sample Wilcoxon rank sum test.

Logistic regression model will be used to explore the efficacy (*QZQG* group and placebo group) and factors affecting efficacy. Dependent variable is the proportion of clinical improvement at day 7. In addition to drugs, independent variables also include the basic information and life behavior factors of participants. The basic information includes gender, height, weight, age, and underlying diseases that include chronic obstructive pulmonary disease, asthma, diabetes, hypertension, hyperlipidemia, hyperuricemia, cardiovascular disease, autonomic nervous dysfunction, lung cancer, other tumors, etc. Life behavior factors mainly include smoking and drinking.

For the efficacy analyses of quantitative variables, comparisons between groups will use repetitive measure analysis of variance and covariance analysis. For qualitative variables, comparisons between groups will use the Pearson’s *χ*^2^ test and center effect analysis will use the Cochran-Mantel-Haenszel (CMH) test. For rating variables, group comparisons will be tested by the Kruskal-Wallis test and the central effect analysis will be tested by the CMH test or grade logistic regression. For center effect analysis, the generalized linear model (GLM) and CMH methods will be used for quantitative and qualitative indicators, respectively. A logistic regression model will be performed for the evaluation and correction of rating variables. For safety analysis, the prevalence of adverse events in the two groups will be compared using the Pearson’s *χ*^2^ test, as well as listing and describing the events that occur during the trial. A description of laboratory test results, electrocardiograph, and chest radiograph will be described as normal/abnormal changes, as well as the relationship between the abnormal changes and the test drug; these changes will be stated.

### Methods for any additional analyses {20b}

Subgroup analysis of all endpoints will be conducted based on chronic respiratory diseases (with or without), complications (with or without) and course of disease (< 3 days and ≥ 3 days). Subgroup analyses will be produced where appropriate and where the number of patients in each subgroup is sufficient.

### Definition of analysis population relating to protocol nonadherence {20c}

The intent-to-treat (ITT) population is defined as all randomized patients, whether or not the patient received the assigned treatment. The ITT patients will be analyzed according to the treatment assigned at randomization. The ITT population includes the patients who agree to enroll in the study and sign an informed consent form. However, ITT population involves those who have never received study drugs or never completed observation records. Efficacy analyses will be conducted for the modified intent-to-treat (mITT) population. This is defined as all patients randomized who received at least one dose of *QZQG* formula and were centrally assessed as RT-PCR positive for influenza at any timepoint, with patients grouped according to the treatment assigned at randomization. The per-protocol set is used to analyze the main outcome for evaluating efficacy and to examine the consistency of the results from the mITT. The PPS is defined as follows: (1) full compliance with inclusion and exclusion criteria; (2) the compliance of medication consumption is over 80%; (3) the patients complete the test and record of the outcome and safety evaluation at every time node; and (4) completion of the clinical trial without major protocol violation. The safety analysis set (SAS) is defined as patients who received any amount of study treatment. Patients will be analyzed according to the treatment received. Patients who were randomized to the study but who did not receive any study drug will not be included in the safety population. The SAS will be used for the analysis of all safety indicators. Multiple imputation will be used for missing data.

## Composition of the coordinating center and trial steering committee {5d}

The study is led by Longhua Hospital Affiliated to Shanghai University of Traditional Chinese Medicine as coordinating center. The data management team is Department of Epidemiology, School of Public Health, Fudan University. The trial steering committee consists of the following members:

1. Pei-yong Zheng, Director of clinical research center of Longhua Hospital Affiliated to Shanghai University of Traditional Chinese Medicine
2. Zhen-hui Lu, Director of the Institute of Respiratory Diseases, Longhua Hospital, Shanghai University of Traditional Chinese Medicine
3. Hui-yong Zhang, Director of lung disease department of Longhua Hospital Affiliated to Shanghai University of Traditional Chinese Medicine
4. Ming Yang, Director of GCP office of Longhua Hospital Affiliated to Shanghai University of Traditional Chinese Medicine
5. Zhi-jie Zhang, Associate professor of Department of Epidemiology, School of Public Health, Fudan University

## Monitoring

### Description of the data monitoring committee {21a}

The independent Data Monitoring Committee (DMC) DMC is responsible for reviewing the reports regarding protocol adherence and making recommendations to continue or terminate the study. The DMC members are all independent of the sponsor/funders and have no financial or other conflicts of interest.

### Harms {22}

#### Safety assessment

The dosage of *QZQG* formula used in this trial is within the recommended range based on the People’s Republic of China Pharmacopeia (2020 edition). To our knowledge, no serious adverse events of this formula have been reported so far. Considering that TCM may cause allergic reactions and gastrointestinal reactions (nausea, vomiting, diarrhea, etc.), it is necessary to arrange clinical tests to evaluate the safety of the formula and protect the participants. Safety is evaluated by using vital signs, cardiopulmonary signs, electrocardiography, chest radiograph, laboratory tests, and adverse events. Vital signs include height, weight, heart rate, blood pressure, and finger pulse oxygen saturation. Laboratory tests include blood routine test, liver and kidney function, electrolyte test, arterial blood gas analysis, bleeding and coagulation function, and myocardial enzyme. The abovementioned and other unmentioned laboratory tests need to be arranged flexibly according to the condition of the participants. But the laboratory tests arranged at the planned visit are necessary.

#### Assessment of adverse events

Any adverse event that occurs to a participant will be recorded in the CRF regardless of relationship to the intervention. Adverse events are divided into three levels: mild, moderate and severe (Table 3). And the adverse events are not necessarily related to the drugs. Any cause-and-effect relationship between adverse events and the study drugs will be determined by the classifications as shown in Table 4. If patients with mild or moderate adverse events do not show significant improvement after medical treatment or drug reduction, the research doctors need to report to the ethics committee and terminate the trial. All serious adverse events will be reported within 24 h to the Principal Investigator, Steering Committee, IRB, the sponsor, and CFDA.

**Table 3:** Classification of disease severity

Classification	Features
**1. Mild**	Participants can continue to participate in the trial without medical treatment and impact on the health.
**2. Moderate**	Participants could not tolerate the drug or need medical treatment. Such events partially affect the participants’ functional activity without threat to life safety.
**3. Severe**	The events pose threat the life of participants, leading to death or disability, which requires immediate withdrawal of medicine or emergency monitoring treatment.

**Table 4:** Classification of the correlation between adverse events and drugs

Classification	Features
**1. Definite**	Use of the experimental drug has a definite relationship with time.
	The event is consistent with the known adverse reactions of Chinese herbal medicine.
	Adverse events disappear after drug withdrawal and reappear after repeated administration.
**2. Probable**	Use of the experimental drug has a reasonable relationship with time.
	Adverse events are partially in line with the known adverse reactions of Chinese herbal medicine.
	It is difficult to identify a cause owing to disease or other reasons.
**3. Possible**	Use of the experimental drug has a reasonable relationship with time.
	Adverse events do not conform to adverse reactions of Chinese herbal medicine.
	The adverse events are likely to be caused by diseases or other reasons.
**4. Remote**	There is a possible connection between time and experimental drug.
	It is easily explained and verified through the disease or other reasons.
**5. Unrelated**	There is no connection between time and the test drug.
	Adverse events are definitely caused by the disease or the main cause of the disease

Participants may experience exacerbations due to natural disease progression, poor drug efficacy, or other reasons, including but not limited to respiratory failure, acute necrotizing encephalopathy, shock, multiple organ dysfunction, and other conditions that require clinical monitoring. If the participant is judged to be critically ill in the process of trial, the research doctor at the center where the participant is located should report to the sponsor and the ethics committee within 24 h. The trial drug intervention should be stopped at the same time. The research doctor should immediately initiate a clinical treatment regimen on severe clinical situation. When necessary, the sponsoring unit and the project funding department will organize multidisciplinary consultations and propose a comprehensive clinical treatment plan. The branch center and the sponsor are jointly responsible for the treatment, follow-up monitoring, and follow-up of critically ill participants.

### Frequency and plans for auditing trial conduct {23}

The supervision team directly led by PI will be responsible for monitoring the trial. On-site monitoring and remote monitoring visits will be conducted in accordance with the study monitoring plan to ensure the completeness and accuracy of research data. Audits may be conducted at any time during or after the study.

## Ethics and dissemination

### Research ethics approval {24}

The IRB of Longhua Hospital Affiliated to Shanghai University of Traditional Chinese Medicine has approved the protocol (IRB approval No.2019LCSY017).

### Protocol amendments {25}

Any protocol amendments will be prepared by the PI. Protocol amendments will be submitted to the IRB and to regulatory authorities in accordance with local regulatory requirements. Approval must be obtained from the IRB and regulatory authorities (as locally required) before implementation of any changes, except for changes necessary to eliminate an immediate and direct hazard to patients, in which case the IRB will be informed as soon as possible.

### Consent or assent {26a}

The investigators, doctors, and other officers in the trial will obtain informed consent or assent from potential trial participants or authorized surrogates. The investigators must issue informed consent to potential participants or authorized surrogates and fully inform the benefits, risks and precautions of participating in this trial. The informed consent form must be signed and dated by trial participants or authorized surrogates.

### Confidentiality {27}

The PI maintains confidentiality standards by coding each patient enrolled in the study through assignment of a unique patient identification number. This means that patient names are not included in data sets that are transmitted to any Sponsor location. Medical information of patients in this study is confidential and may be disclosed to third parties only as permitted by the Informed Consent Form (or separate authorization for use and disclosure of personal health information) signed by the patient, unless permitted or required by law. Medical information may be given to a patient’s personal physician or other appropriate medical personnel responsible for the patient’s welfare, for treatment purposes.

### Access to data {29}

Statement of who will have access to the final trial data set, and disclosure of contractual agreements that limit such access for investigators.

### Dissemination plans {31a, 31b, 31c}

We will communicate trial results to national and international health authorities, healthcare professionals, the public, and other relevant groups as soon as the trial results are available. There is currently no plan to disclose public access to the full protocol, participant-level data set, and statistical code.

## Trial registration {2a, 2b}

We registered our protocol (ChiCTR2000028708) on the Chinese Clinical Trial Registry (ChiCTR) on 1 January 2020, which is one of the primary registries of the World Health Organization International Clinical Trials Registry Platform. All items from the World Health Organization Trial Registration Data Set requirements are met with the trial’s registration in the ChiCTR.

## Protocol version {3}

The protocol version is number 1.0, dated October 10, 2018.

## Discussion

Severe influenza tends to result in critical illness and sometimes death compared with mild influenza [4, 6]. Oseltamivir, as one of the most important antivirals widely used in influenza, has several limitations including a short therapeutic time window, a low genetic barrier to resistance, limited antiviral efficacy [29], and, most importantly, uncertainty regarding its effectiveness in severe influenza [30, 31]. While Chinese herbal medicine mainly based on TCM theory is widely used in influenza in China, currently there is not enough clinical evidence to evaluate whether TCM is effective and safe for treating severe influenza [2]. The quality of many prior studies on TCM was assessed to be generally low due to methodological limitations such as inadequate randomization, lack of double blinding and non-placebo control, incomplete outcome data [32]. Therefore, this protocol is rigorously-designed in accordance with the SPIRIT statement, CONSORT statement, and CONSORT Extension for Chinese Herbal Medicine Formulas [33–35]. To our knowledge, this study is the first randomized double-blind placebo-controlled trial of TCM as an adjuvant therapy for adult patients with severe influenza.

Despite no RCTs to support clinical outcomes benefit with antiviral drugs in the severe influenza, prompt initiation of NAIs was recommended as the mainstay of antiviral treatment in severe influenza [9]. Therefore, adults with severe influenza in this trial need to receive oral oseltamivir in addition to necessary hospitalized intravenous treatment. The use of placebo will help to objectively evaluate the true effect of drugs when the pressure of doctors to make decisions based on preliminary but inconclusive data is intense. A placebo and double-binding can reduce the impact of subjective thoughts and conflicts of interest from researchers and participants. Under the situation of COVID-19 global pandemic, many hospitals participating in this trial need to undertake huge pressure and tasks. And it is pretty difficult to carry out such a large clinical trial. We need to resist such pressures and make sure that patients benefit from the fruits of science, even in difficult times.

The choice of the primary outcome for RCTs is important and challenging for the rational evaluation of the test drug. Adults with severe influenza have various clinical outcomes, including discharge, hospitalization without oxygen, hospitalization requiring respiratory support or ECMO, and even death. We believe that a single end point (the duration of viral shedding, the time to clinical symptom alleviation, etc.) may not accurately assess the effect of the test drug. Therefore, 7-category ordinal scale and NEWS2 as composite clinical outcome used in severe influenza trial before [26, 36] is required for the evaluation of the efficacy of *QZQG*, which is both pragmatic and informative [37]. At the same time, a range of secondary endpoints to show consistency with the primary endpoint have been included in this trial, as recommended by the US Food and Drug Administration for trials in influenza [38].

## Supplementary Information

**Additional file 1.**

## Abbreviations

CMH: Cochran-Mantel-Haenszel
CONSORT: Consolidated Standards of Reporting Trials
COVID-19: Coronavirus disease 2019
CRF: Case report form
DMC: Data Monitoring Committee
ECMO: Extracorporeal Membrane Oxygenation
FAS: Full analysis set
GLM: Generalized linear model
ICU: Intensive care unit
IRB: Institutional review board
ITT: Intent-to-treat
mITT: Modified intent-to-treat
NAIs: Neuraminidase inhibitors
NEWS2: National Early Warning Score 2
PP: Per-protocol
*QZQG* formula: *Qiangzhu-qinggan* formula
RCT: Randomized controlled trial
SAS: Safety analysis set
SPIRIT: Standard Protocol Items: Recommendations for Interventional Trials
TCM: Traditional Chinese medicine
