# Supplementary material for: Evaluation of efficacy and safety of Qiangzhu-qinggan formula as an adjunctive therapy in adult patients with severe influenza: study protocol for a randomized parallel placebo-controlled double-blind multicenter trial
Source: Trials. 2021 Dec 27;22:955. doi: 10.1186/s13063-021-05929-8 (PMC8710932; doi:10.1186/s13063-021-05929-8)
Supplement: Supplementary file 1 — Additional file 1. [file 13063_2021_5929_MOESM1_ESM.pdf]

Informed Page

Dear patient:

Your doctor has confirmed that you have severe influenza and we will invite you to participate in a clinical study: Evaluation of efficacy and safety of Jiuweiqianghuo-Zhuyeshigao formula as an adjunctive therapy in adult patients with severe influenza: study protocol for a randomized parallel placebo-controlled double-blind multicenter trial. This is a randomized, controlled, parallel, double-blind clinical trial to evaluate the efficacy and safety of traditional Chinese herbal formula granules as an adjuvant therapy in adult patients with severe influenza. This study is supported by the Project of the Science and Technology Commission of Shanghai China (18495810600), Major clinical research project of Shanghai Shenkang Hospital Development Center (SHDC2020CR2006A) and the National Major Infectious Disease Project of the Ministry of Science and Technology of China (2017ZX10305501002; 2018ZX10725-509). The IRB of Longhua Hospital Affiliated to Shanghai University of Traditional Chinese Medicine has approved the protocol (IRB approval No.2019LCSY017).

Before you decide whether to participate in this study, please read the following as carefully as possible. It can help you understand the study and why you want to conduct the study, the procedure and duration of the study, and the benefits, risk and discomfort that may be brought to you after participating in the study. If you wish, you can also discuss with your relatives and friends, or ask your doctor to explain and help you make a decision.

- **Background**

Influenza can fall into three categories according to severity: mild influenza, severe influenza, and critical influenza. Severe influenza can result in critical illness and sometimes death particularly in patients with comorbidities, advanced age, or pregnancy. Neuraminidase inhibitors (NAIs) are the only antiviral drugs in widespread use for influenza. However, the effectiveness of NAIs against severe influenza is uncertain. New effective drugs or regimens are therefore urgently needed. Jiuweiqianghuo-Zhuyeshigao formula has been found to be effective against influenza virus infection during long-term application in China, which lacks support of evidence-based clinical trial till now. This study is designed to assess the efficacy and safety of JWQH-ZYSG as an adjuvant therapy in adult patients with severe influenza.

- **Objectives**

Empirical evidence based on long-term clinical application shows that JWQH-ZYSG formula can effectively resist influenza virus infection. However, there are currently no reliable data from evidence-based clinical trials. We aim to carry out this trial to gain a deeper insight into the efficacy and safety of JWQH-ZYSG formula in adults with severe influenza.

- **Study setting**

This trial will be conducted in Longhua Hospital Affiliated to Shanghai University of Traditional Chinese Medicine, Huashan Hospital Affiliated to Fudan University, The First Affiliated Hospital of Anhui University of Traditional Chinese Medicine, Hefei Infectious Disease Hospital, Jiangsu Provincial Hospital of Traditional Chinese Medicine, Jiangsu Provincial Hospital for Infectious Diseases and Zhejiang Provincial Hospital of Traditional Chinese Medicine. Prior to the trial, all personnel are trained in Longhua Hospital to ensure that the physicians and staff participating in the trial at every center fully understand all aspects of the trial

- **Who are not suitable for the study?**

(1) Age<14 years or >65 years.

(2) Participants hospitalized for exclusively social reasons (e.g., lack of caregivers at home).

(3) Participants expected to be discharged within 48 hours, according to the investigator's judgement.

(4) Participants weighing < 40 kg.

(5) Participants who have received more than 48 hours of antiviral treatment for the current influenza infection prior to screening.

(6) Participants with known severe renal impairment or receiving continuous renal replacement therapy, hemodialysis, peritoneal dialysis.

(7) Participants with any of the following laboratory abnormalities detected within 24 hours prior to or during screening (according to local laboratory reference ranges:

Alanine Transaminase (ALT) or Aspartate Transaminase (AST) level > 5 times the upper limit of normal (ULN).

ALT or AST > 3 times the ULN and total bilirubin level > 2 times the ULN.

(8) Any serious medical condition or abnormality in clinical laboratory tests that, in the investigator's judgment, precludes the participant's safe participation in and completion of the study.

(9) With mental changes or convulsions (e.g., slow response, drowsiness and restlessness).

(10) With severe gastrointestinal symptoms (e.g., severe nausea, vomiting, diarrhea and even dehydration).

(11) With one of the following critical illnesses: respiratory failure, acute necrotic encephalopathy, septic shock, multiple organ dysfunction, or other serious clinical conditions requiring intensive care.

(12) With tuberculosis, measles, AIDS or other infectious diseases.

(13) Women who are pregnant (including a positive pregnancy test at enrollment), breastfeeding, or within 2 weeks post-partum.

(14) Known history of allergy or severe intolerance of oseltamivir and herbal medicines, as determined by the investigator.

(15) Currently or have been involved in another anti-influenza treatment trial in the last 28 days.

(16) Patients who have been treated with oral Chinese medicine within 4 weeks.

(17) Patients who, in the opinion of the investigator, would be unlikely to comply with required study visits, self-assessments, and interventions.

What would you need to do if you took part in the research?

(1) Before you are enrolled in the study, your medical history will be asked and recorded, and you will be given a physical examination to determine whether you can participate in the study.

(2) If you meet the inclusion and exclusion criteria, the study will be conducted according to the following steps:

→ At the beginning of the study, you will receive the treatment group (oseltamivir + *JWQH-ZYSG* Granules) or the control group (oseltamivir + placebo) according to the random number provided by the computer. Participants had a 50% chance of being divided into these two groups. The treatment of *JWQH-ZYSG* Granules lasted for 1 week.

→ The doctor will check your vital signs and various systems every day, record symptom scores and monitor changes in body temperature, and require the following checks according to your condition: Viral nucleic acid testing, blood routine, urine routine, liver function, kidney function, blood coagulation function, heart function, immune function and other laboratory tests.

(3) Other matters requiring your cooperation: You must follow the instructions of your doctor to take the medicine. During the study period, you cannot use any other traditional Chinese medicine for severe influenza. If you need other treatments, please contact your doctor in advance.

- **Possible benefits of participating in research**

You and society will likely benefit from this research: your condition may be improved, and a new treatment method may be discovered in this topic for other patients with similar conditions.

- **Possible adverse reactions and risks of participating in the study**

Adverse reactions such as nausea, vomiting, and diarrhea may occur. If there is an adverse reaction in the study, regardless of whether it is related to the drug, your doctor will make judgment and medical treatment. The doctors and sponsors will do their best to prevent and treat the possible harm caused by this study. If an adverse event occurs in a clinical trial, the investigator will identify whether it is related

to the trial drug. The sponsor will provide corresponding treatment costs for the damages clearly related to the trial drug, which has been stipulated in Good Clinical Practice in China.

- **Explanation of fees**

The sponsor of this study will pay for the research-related inspections you have done during the study period and provide study medication for free. After the study is over, you will receive transportation compensation for participating in the clinical trial. If damages related to the trial drug occur, the sponsor will pay you the corresponding medical expenses. If you combine the treatment and examination required for other diseases at the same time, it will not be included in the free scope.

- **Is personal information confidential?**

Your medical records (research medical records/CRF, inspection reports, etc.) will be kept in the hospital. Investigators, sponsor representatives, ethics committees, and drug regulatory authorities will be allowed to access your medical records. Any public report on the results of this research will not disclose your personal identity. We will make every effort to protect the privacy of your personal medical data within the scope permitted by law.

- **How can I get more information?**

You can ask any questions about this study at any time, and your doctor will answer your questions carefully.

- **About voluntary participation and dropout research**

Whether to participate in the study is entirely up to your volition. You can refuse to participate in this study, or withdraw from this study at any time during the study process, this will not affect your medical benefits. If you withdraw from the study for any reason, you may be asked about your use of the trial drug. You may also be asked for laboratory tests and physical examinations if the doctor thinks it is necessary. If you do not participate in this study, or withdraw from the study, there are many other alternative flu intervention drugs. If you choose to participate in this research, we hope that you will continue to complete the entire research process.

- **What should you do now?**

Whether to participate in this study is up to you. You can make a decision after discussing it with your family or friends. Before you make a decision to participate in the study, please ask your doctor about the relevant questions as much as possible until you fully understand the study.

Thank you for reading the above materials. If you decide to participate in this study, please tell your doctor and he will arrange all matters related to the study for you.

Please keep this informed consent form.

Consent signature page

Name: Evaluation of efficacy and safety of Jiuweiqianghuo-Zhuyeshigao formula as an adjunctive therapy in adult patients with severe influenza: study protocol for a randomized parallel placebo-controlled double-blind multicenter trial.

Responsible unit: Longhua Hospital Affiliated to Shanghai University of Traditional Chinese Medicine

Ethics review batch number: 2019LCSY017

Consent statement:

1. I have read the above introduction about this research and have the opportunity to discuss and ask questions about this research with the doctor. All the questions I raised have been answered satisfactorily.
2. I know the risks and benefits that may arise from participating in this research. I know that participating in the research is voluntary.
3. I confirm that I have sufficient time to consider this and understand the following items:

→ I can always consult the doctor for more information.

→ I can withdraw from this study at any time without being discriminated against or retaliated, and medical treatment and benefits will not be affected.

→ I understand that if I withdraw from the study halfway, I should inform the doctor of the changes in my condition and complete the corresponding physical examination and physical and chemical examination. This will be very beneficial to me and the whole study.

→ I agree to the health department, ethics committee or professional academic committee to review my research materials.

→ I will get a signed and dated copy of the informed consent.

Finally, I decided to agree to participate in this research and try to follow the doctor's advice

Participant's signature: Signature of the participant's agent:

Date of signature: Contact details:

Doctor's Statement

I confirm that I have explained to the patient the details of the study, including its rights and possible benefits and risks, and gave him or her a copy of the signed informed consent form.

Doctor's signature:

Date of signature: Contact details:
